# Psychometric evaluation of the Interpersonal Needs Questionnaire (INQ) using item analysis according to the Rasch model

**DOI:** 10.1371/journal.pone.0232030

**Published:** 2020-08-03

**Authors:** Luz Dary Upegui-Arango, Thomas Forkmann, Tine Nielsen, Nina Hallensleben, Heide Glaesmer, Lena Spangenberg, Tobias Teismann, Georg Juckel, Maren Boecker

**Affiliations:** 1 Institute of Medical Psychology and Medical Sociology, University Hospital of RWTH Aachen University, Aachen, Germany; 2 Department of Clinical Psychology, University of Duisburg-Essen, Essen, Germany; 3 Department of Psychology, University of Copenhagen, Copenhagen, Denmark; 4 Department of Medical Psychology and Medical Sociology, University of Leipzig, Leipzig, Germany; 5 Mental Health Research and Treatment Center, Department of Psychology, Ruhr-Universität Bochum, Bochum, Germany; 6 Department of Psychiatry, LWL-University Hospital, Ruhr-Universität Bochum, Bochum, Germany; eCampus University, ITALY

## Abstract

The Interpersonal Needs Questionnaire (INQ) assesses Thwarted Belongingness (TB) and Perceived Burdensomeness (PB), two predictors of suicidal thoughts. Up to now, the use of item response theory (IRT) for the evaluation of the INQ has been restricted to a single study with clinically depressed and suicidal youth. Therefore, the psychometric properties of the two INQ-15-subscales TB and PB were now evaluated in a general population sample (N = 2508) and a clinical adult population sample (N = 185) using IRT, specifically the Rasch model (RM) and the graphical log-linear Rasch model (GLLRM). Of special interest was whether the INQ-subscales displayed differential item functioning (DIF) across the two different samples and how well the subscales were targeted to the two sample populations. For the clinical sample, fit to a GLLRM could be established for the PB-subscale and fit to a RM was established for a five-item version of the TB-subscale. In contrast, for the general population sample fit to a GLLRM could only be achieved for the PB-subscale. Overall, there was strong evidence of local dependence (LD) across items and of some age- and gender-related DIF. Both subscales exhibited massive DIF related to the sample, indicating that they don’t work the same across the general population and clinical sample. As expected, targeting of both INQ-subscales was much better for the clinical population. Further investigations of the INQ-15 under the Rasch approach in a large clinical population are recommended to determine and optimize the scale performance.

## Introduction

The number of suicides is constantly increasing worldwide, with a global increase of 6.7% (CI 95%: 0.4% -15.6%) between the period of 1990 and 2016 (817000 deaths in 2016). [1] Even though, historically, the majority of suicides have been reported in persons being aged 70 years or more (WHO, 2014), the number of cases for young individuals has been rising. Nowadays, suicide is the second leading cause of death in the population of people aged 15 to 29 years. [2] Despite the significant public health implications of suicide, there are only few countries that have introduced national prevention strategies. The main reasons for the absence of national prevention strategies seem to be due to a lack of awareness for the importance of the problem, lack of social discussion of the issue due to stigma. [3]

Recent meta-analytic evidence showed that the prediction of suicidal behavior and suicidal deaths is weak, although the number of studies in the field is constantly growing. [4] One of the main implications of the results of this meta-analysis is that more research should be guided by modern theories within an ideation-to-action-framework. [5] One of the most intensively investigated ideation-to-action models is the “Interpersonal Theory of Suicide” (IPTS), which suggests a three-dimensional model that explains how suicidal ideation develops and why people transit from suicidal ideation to plans and behavior. [6] The theory proposes three core constructs, of which two predict the desire for suicide, Thwarted Belongingness (TB) and Perceived Burdensomeness (PB) while the third construct refers to the capability of realizing the act of suicide and predicts the transition from ideation to action. [7] TB refers to the sense of not belonging to important groups like family and society, where the individual responds negatively to statements such as (“these days, other people care about me”). PB refers to the view on oneself as being a burden to others and it is reflected by perceptions such as (“These days the people in my life would be better off if I were gone”). [7] The Interpersonal Needs Questionnaire (INQ) was designed based on classical test theory (CTT) to measure these two central components of the IPTS. [8] Several versions of the INQ with 10, 12, 15, 18 and 25 items have been developed, but the 10-item version and 15-item version have evidenced the best model fit in confirmatory factor analyses (CFA). [9] The 10-item version mostly utilized among military samples, and the 15-item version is an empirically derived refinement of the INQ-25 used in a variety of different samples. [10]

Until today, there are only very few studies that have evaluated the psychometric properties of the INQ 15-item version, and these evaluations were done using CTT approaches. In these studies, convergent validity and good reliability have been demonstrated as well as the two-dimensional structure of the instrument (TB and PB). [9,11,12] A German translation of the INQ-15 was previously published. [13] Based on a representative sample of the German general population (n = 2513) and using CTT, the two-factorial structure of the INQ-15 was evidenced through confirmatory factor analysis, very good internal consistencies (α ≥0.89) were reported and normative data were provided. [11]

Modern test approaches like item response theory (IRT) have had a limited application for evaluating the INQ, despite their methodological advantages compared with CTT, especially in high-stake situations. [14] For instance, El-Behadli and colleagues evaluated the INQ 25 in clinically depressed and suicidal youth using the Rasch model (RM). They proposed a 10-item version with 5 items in each subscale and with response categories reduced from 7 to 4 options. [15] Although they evaluated aspects such as unidimensionality and item fit, they did not evaluate local dependence (LD), differential item functioning (DIF) and the targeting of the INQ subscales. This could limit the interpretation of the findings of El-Behadli and colleagues and the application of the proposed INQ-10 version, since the presence of items with LD or DIF could lead to estimating biased parameters. [16–18] Additionally, as targeting was not assessed, the suitability of the scale range was not established within the investigated population.

Thus, the aim of the present study was to use IRT to evaluate the psychometric properties of the German INQ15 version in the general and a clinical population. Therefore, the data of the German INQ-validation study (Hallensleben et al., 2016) were re-analysed, this time using the IRT approach. [11] As the validation study had been based upon a representative sample of the German population, which did not include clinical data, additional data of a clinical population were added. [19–23] The clinical population mainly consisted of patients suffering from an affective disorder according to ICD-10, and patients who were admitted to hospital because of suicidal tendencies. Having these two very different types of samples in the present study, it was hypothesized that the TB and PB subscale would perform better in the clinical population, since the viability of the representation of the latent structure of the INQ has been previously established within a clinical sample. [10] Moreover, it was also predicted that the items of the INQ might not work the same in clinical and general populations, i.e. that there might be DIF in some INQ items with respect to the sample. Therefore, a special focus was directed towards the investigation of DIF as well as targeting. Investigating the targeting will provide evidence relating to the measurement information provided by the two subscales, and whether sufficient measurement precision is achieved.

## Materials and methods

### Research design

Cross sectional study using Rasch analysis.

### Participants

The general population sample composed 2508 participants and the clinical sample 185 participants. The general population was a representative sample of the German population and was part of a larger study where data was collected from 258 areas of Germany. [11] Inclusion criteria were age ≥14 years and good skills in German language. The questionnaire was completed by the subjects who had agreed to participate following informed consent, including from one parent where the respondent was a minor. 55.5% of the sample were females and the mean age was 49.2 (SD = 18) (see Table 1 for a more detailed sample description). The study was approved by the ethics committee of the Faculty of Medicine of the University of Leipzig.

**Table 1:** Characteristics of the study groups.

	GP n = 2508 (%)		Clinical n = 185 (%)	Mixed1 n = 370 (%)	Mixed2 n = 370 (%)
**Age Group**^**a**^		**Age Group**^**b**^			
≤34 years	652 (26.0)	≤26 years	47 (25.4)	95 (25.7)	94 (25.4)
35 to 49 years	611 (24.4)	27 to 36 years	48 (25.9)	97 (26.2)	96 (25.9)
50 to 63 years	653 (26.0)	37 to 48 years	43 (23.2)	87 (23.5)	86 (23.2)
≥64 years	592 (23.6)	≥49 years	44 (23.8)	88 (23.8)	91 (24.6)
**Average Age (SD)**	48.79 (18.10)		37.87 (13.07)	37.68 (13.53)	38.59 (14.82)
**Gender**					
Male	1117 (44.5)		88 (42.9)	158 (42.7)	163 (44.1)
Female	1391 (55.5)		97 (57.1)	211 (57.0)	206 (55.8)

**GP:** general population; **Clinical**: clinical population; **Mixed1:** general population (n = 185 age- and gender-matched random sampling without PB extreme scores of the general population) and clinical sample (n = 185); **Mixed2:** general population (n = 185- age- and gender-matched random sampling without TB extreme scores of the general population) and clinical sample (n = 185).

^a^ Based on general population age distribution.

^b^ Based on clinical sample age distribution.

The data of the clinical population were collected within two larger multicenter studies on the course and prediction of suicidal ideation and behavior that have been conducted at the Universities of Aachen, Bochum and Leipzig. [19–23] Data were collected in nine different psychiatric and psychosomatic clinics. All participants provided written informed consent prior to joining the study. The AMBAS study was approved by the ethics committee of the Medical Faculty of the University of Leipzig (No: 388-13-16122013) and the PRESS study by the ethics committees of the Medical Faculty of the RWTH Aachen University (No. EK 310/13), and of the Universities of Bochum and Leipzig. Fifty-two percent of the participants were women and the mean age was 38 years (SD = 13.3) (Table 1). The sample consisted of 97 patients suffering from depression and 88 patients with suicidal history.

To evaluate age-related DIF, the age variable was categorized into four groups. Because of the different age structure in the clinical and general population samples the categorization was carried out separately for the two samples. Additionally, two mixed samples were used to investigate whether DIF with regard to sample (general population vs. clinical) could be found while controlling for the different sample sizes and for potential age- or gender-related DIF-effects. Each was composed of the clinical population and 185 randomly drawn age- and gender-matched people from the general population (see Table 1).

In the composition of the mixed samples, care was taken to ensure that just a few persons from the general population with extreme values were chosen, as extreme values do not contribute to the parameter estimation in the RM framework. However, the percentage of extreme values in the general population sample was much higher for the PB-subscale (86.6%) as compared to the TB-subscale (20.5%). Therefore, besides the mixed1 sample which was drawn for the PB-subscale based on the explained restrictions, a second sample (mixed2) was drawn for the TB-subscale.

### The Interpersonal Needs Questionnaire

The INQ-15 is a self-report questionnaire consisting of 15 polytomous items. [10] The TB dimension and PB dimension are represented by 6 and 9 items respectively, with the response options ranging from 1 (not at all true for me) to 7 (very true for me) (see S1 Table in S1 Material). The items 7, 8, 10, 13, 14, and 15 of the TB subscale are reversely coded. Thus, higher scores reflect higher levels of TB and PB. Previously, the translation and cultural adaptation of the INQ into a German version was implemented. [13]

### Statistical analysis

The descriptive analysis was performed using the software SPSS version 22 and the DIF-equated score graphics with Stata version 14. [24,25] The item analysis according to the RM was carried out using the software DIGRAM 4.09.05. [26,27]

### The Rasch model and graphical loglinear Rasch models

An IRT model with especially desirable mathematical properties is the RM. [14] It is one of the parsimonious IRT models in mathematical terms, which permits to investigate the item and persons characteristics independently, as well as the relationship between these parameters. [28] Furthermore, the RM not only produces interval-level person estimates, together with an individual standard error of measurement for each person (which depends on the person’s location on the continuum), but also its findings are independent from both the sample and scale (specific objectivity), together with sufficiency of the sum scores. These two last properties are exclusively the result of fit to the RM. [29] In contrast to CTT, IRT does not assume that the measurement precision is constant across the scale, but depends on where the items are located and where they provide enough information to differentiate between persons. [14,30]

The five following aspects of measurement have to be fulfilled in order to meet RM requirements:

1. Unidimensionality: The items of the scale assess one single underlying latent construct.
2. Montononicity: The expected item scores have to increase with increasing values of the latent construct.
3. Absence of LD: The response to a single item should be conditionally independent from the response given to another item of the scale given the latent trait.
4. Absence of DIF: Items should be equally difficult to endorse across people of different subgroups based on exogenous variables (e.g. gender or age group) given the latent trait.
5. Homogeneity: The rank order of the item parameters is the same across all persons regardless of their level on the latent trait.

Departures from the RM in form of DIF or LD are often found for scales. If this is the case, the graphical loglinear RM (GLLRM) belonging to the family of RMs, can be used. [31] It allows for departures from the RM in form of uniform DIF and/or uniform LD in case the RM is rejected. [31,32] In the GLLRM the LD and DIF terms are added as interactions terms in the model. GLLRMs still have most of the desirable properties of the RM, and the sum score will still be a sufficient statistic in case that an adjustment due to LD has to be undertaken.

### Item analysis according to the RM and the GLLRMs

Seven lines of analyses were performed to check whether the INQ data met the assumptions of the RM or in case of no fit the assumptions of the GLLRM. [31] The polytomous RM was used. [33,34] Analyses were done separately for the two subscales PB and TB. Three separate analyses were run for the PB-subscale: 1. for the clinical sample, 2. for the general population sample, and 3. for a mixed sample of the clinical sample and the general population (mixed1), where the extreme scores of the general population were excluded (n = 370). For the TB-subscale four separate analyses were performed: 1. for the clinical sample, 2. for the general population, 3. for the mixed1 sample and, 4. for a second mixed sample of the clinical sample and the general population (mixed2). In this latter sample the extreme scores of the general population were excluded according to extreme scores in the TB-subscale (n = 370).

For each line of analyses, the same general strategy was used as an iterative process: Initially, fit to the RM was tested. In case the RM was rejected, and the departures consisted of LD and/or DIF, fit to a GLLRM accounting for these departures was tested. If fit to a GLLRM could not be established, it was investigated whether the elimination of the most problematic item would improve fit to a GLLRM. Finally, a new cycle of analysis with the remaining items was performed.

In doing so, each analysis included several tests. For the global test-of-fit which assesses item homogeneity across high and low scoring groups as well as for the global test-of-no-DIF Andersen’s conditional likelihood ratio test (CLR) was used. [35] Individual item fit statistics were evaluated by comparing the observed and expected correlations between the score for each item with the total score for all other items (rest scores). [36] To assess LD and DIF in the GLLRMs, conditional tests of independence were applied, using partial Goodman-Kruskal gamma coefficients for assessing the conditional association between item pairs (presence of LD) or between items and exogenous variables (presence of DIF) given the rest-scores. [37] Additionally, after achieving fit to a GLLRM which included interaction terms for the discovered DIF and LD, confirmatory tests of no DIF or LD were used to test whether all interaction terms were needed. This was done using Kelderman’s (1984) likelihood ratio test. In all tests the Benjamini-Hochberg procedure was used as required to adjust the false discovery rate (FDR) due to multiple tests. [38]

In all seven lines of analyses DIF was evaluated related to gender and age groups. In the mixed samples DIF by sample was additionally investigated (general population vs. clinical sample). In case of DIF, the impact of DIF was evaluated by computing equated scores (e.g. Christensen et al., 2019). [39]

The reliability was estimated using the Monte Carlo method proposed by Hamon and Mesbah (2002).[40] In case of model fit, the targeting of the two subscales was evaluated graphically and numerically. For the graphical evaluation, item maps with the distribution of person parameters locations in relation to the distribution of item thresholds were created.[41] For the numerical assessment, two indices with an expected value close to one were applied, the test information target index (mean test information divided by the maximum test information for theta) and the root mean squared error (RMSE) target index (minimum standard error of measurement divided by the mean standard error of measurement for theta). Additionally, the target of the observed score as well as the standard error of measurement (SEM) of the observed score were estimated.

In the case of the TB-subscale, as this subscale is composed of three negatively formulated items and six positively formulated items, unidimensionality was tested (see S1 Table in S1 Material). In doing so, a test of equality was used which compared the observed correlations of the subscores for the positively and negatively formulated items with the subscore correlations expected under the assumption of a unidimensional RM. [42] To obtain exact p-values a Monte Carlo approach with 1000 samples was applied.

## Results

In the following, the results of the item analyses according to the RM and GLLRM are reported separately for the two subscales.

### Item analysis according to the Rasch model: PB-subscale

Fit to the RM couldn’t be found for any of the three different samples for the PB-subscale. Most of the items showed reversed thresholds and there was strong evidence of LD. However, as it was not possible to complete the subsequent GLLRM analyses due to convergence problems, all following analyses had to be done with recoded response categories (from 1234567 to 1223344).

### Clinical population

For the clinical sample fit of the PB to the RM was also rejected after the recoding of response categories (see Table 2). Overall item homogeneity could be confirmed indicating similar item parameters in low and high scoring groups. However, the global test-of-no-DIF showed moderate evidence of age-related DIF.

**Table 2:** PB-subscale (4 response categories): Global test-of-fit and global-tests-of-no-DIF relative to gender, age group and sample.

Sample	Model	homogeneity			DIF relative to									Comment
					gender			age			sample			
		*CLR*	*df*	*p*	*CLR*	*df*	*p*	*CLR*	*df*	*p*	*CLR*	*df*	*p*	
*Clinical*	RM	26.1	17	.073	19.1	17	.321	88.4	51	**.001**				
	GLLRM	43.1	39	.301	33.1	26	.160	64.1	42	.**016**^**a**^				
*GP*	RM	44.3	17	**< .001**	13.4	17	.709	53.7	51	.371				
	GLLRM	37.8	30	.154	36.0	30	.209	108.8	90	.087				
*Mixed1*^§^	RM	47.1	17	**< .001**	10.5	17	.883	64.5	51	.097	117.7	17	**< .001**	No final model could be reached–indication of more LD
	GLLRM	30.3	51	.990	23.1	29	.770	114.0	87	**.028**^b^	3.9	5	.564	

**GP:** general population; **Clinical:** clinical sample. Benjamini-Hochberg adjustment for FDR

^a^ Reject if p-value is < .0167.

^b^ Reject if p-value is < .050.

^§^There was no final GLLRM.

Further inspection showed that this age-related DIF could be traced back to item INQ2 (“These days the people in my life would be happier without me.”; see Table 3). For this item the age groups from 27 to 36 years and from 37 to 48 years tended to systematically score higher given the same level of PB.

**Table 3:** Conditional likelihood ratio tests of DIF under the respective GLLRMs for the PB-subscale.

Item and background variable	CLR	df	p
*Clinical*			
INQ2 & age	23.06	9	**.006**
*Mixed1*			
INQ1 & sample	16.92	3	**< .001**
INQ2 & sample	27.54	3	**< .001**
INQ4 & sample	36.38	3	**< .001**
INQ5 & sample	88.59	3	**< .001**

**Clinical:** clinical sample.

In addition to the age-related DIF for item 2, departures from the RM in form of LD were found (see Table 4). There was strong evidence of LD for the item pair INQ1 (“These days the people in my life would be better off if I were gone”) and INQ2 (“These days the people in my life would be happier without me”), and moderate evidence for the item pair INQ1 and INQ4 (“These days I think my death would be a relief to the people in my life”).

**Table 4:** Conditional likelihood ratio tests of local dependence under the respective GLLRMs for the PB-subscale.

Items in subscales	CLR	Df	p
*Clinical*			
INQ1 & INQ2	73.72	9	**< .001**
INQ1 & INQ4	26.34	9	**.001**
*GP*			
INQ1 & INQ2	64.86	9	**< .001**
INQ4 & INQ5	29.97	9	**< .001**
*Mixed1*			
INQ1 & INQ2	82.91	9	**< .001**
INQ1 & INQ4	40.43	9	**< .001**
INQ4 & INQ5	25.10	9	**.002**

**GP:** general population; **Clinical:** clinical sample.

**Fig 1:**
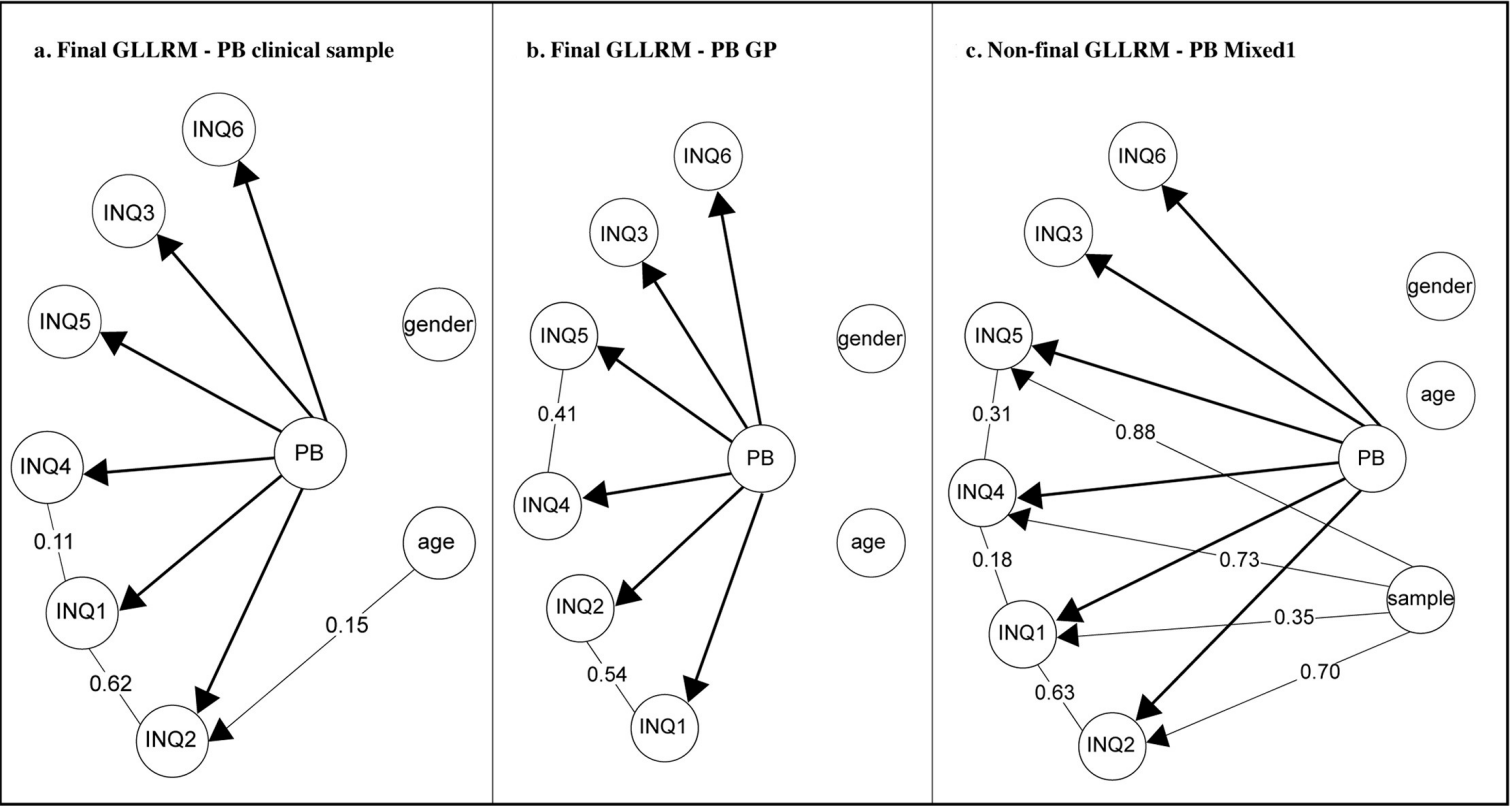
The graphical loglinear Rasch models for the PB-subscale. Lines between items show evidence of LD, arrows pointing from the exogeneous variables to the items evidence of DIF. γ-correlations are partial Goodman and Kruskal’s rank correlations for ordinal data.

After adjusting for DIF and LD, fit to a GLLRM could be found (see Table 2) with no further indication of LD (see S2 Table in S1 Material). The global test-of-no-DIF still showed a weak evidence for additional age-related DIF. However, there was no evidence for age-related DIF for further items (see S4 Table in the S1 Material). The fit of the individual subscale items to the RM and to the final GLLRM is reported in the S6 Table of the S1 Material. Whereas strong evidence of item misfit of item INQ2 was found for the RM, all individual items fitted the final GLLRM. This final model is graphically presented in Fig 1.

In S8 Table in the S1 Material the targeting of the PB-subscale is presented. The person-item threshold distribution maps for the four different age groups of the clinical sample (see S16a Fig in the S1 Material) show that overall the items were well spread along the PB dimension. Most of the person parameters of the clinical sample were located within the same interval as the item parameters. However, especially at the extremes of the dimension being indicative for having no vs. a strong feeling to be a burden, item thresholds were absent which was also reflected by the low test information values in these areas. The test information target index was moderate for the two older age groups and good for the younger ones indicating that between 60% and 76% of the maximum obtainable test information was reached on average for the different age groups. The reliability of the PB-subscale was high for each of the age groups with the weighted mean across the age groups being .89.

Finally, the impact of the observed DIF related to age was evaluated by computing equated scores across the four age groups of the clinical sample. The biggest difference found for the equated scores was .74 (see S10 Table and S15a Fig in the S1 Material).

### General population

Likewise, no fit to the RM could be found for the general population (see Table 2). As distinguished from the clinical sample, there was no evidence of DIF related to age but strong evidence against global homogeneity. Departures from the RM were found in form of strong LD between the item pairs INQ1 (“These days the people in my life would be better off if I were gone”) and INQ2 (“These days the people in my life would be happier without me”) as well as INQ4 (”These days I think my death would be a relief to the people in my life”) and INQ5 (“These days I think the people in my life wish they could be rid of me”). Moderate misfit of individual items was found for items INQ2, INQ3 and INQ4 (see S6 Table in the S1 Material). Accounting for the discovered LD resulted in fit to a GLLRM with no further evidence of LD, DIF or item misfit (see Fig 1, and S2, S4 and S6 Tables in the S1 Material).

The reliability was good (.87). However, as shown in S8 Table and S16b Fig in the S1 Material the targeting for the general population was extremely poor with most people not reporting to have the feeling to be a burden to other people. Indeed, the floor effect was high with 86.5% of the people having the lowest score in the PB-subscale. Beyond that, the poor targeting was reflected by the test information target index which was 0.10. This value indicates that only 10% of the maximum obtainable test information was reached on average for the general population sample.

### Mixed1 sample of general population and clinical population

For the mixed1 sample the overall test-of-fit of the RM rejected item homogeneity. The global test-of-no-DIF suggested no evidence of DIF for gender and age, but strong evidence of DIF for sample (see Table 2). And indeed, considerable DIF relative to sample was found for the following four items: INQ1 (“These days the people in my life would be better off if I were gone”), INQ2 (“These days the people in my life would be happier without me”), INQ4 (“These days I think my death would be a relief to the people in my life”) and INQ5 (“These days I think the people in my life wish they could be rid of me”) (Table 3). For all items the people from the clinical sample tended to answer systematically higher than the people from the general population at the same level of PB (see S11, S14 Tables and S15b Fig in the S1 Material). In addition to the DIF, strong evidence of LD was found for the item pairs INQ1 and INQ2, INQ1 and INQ4 as well as INQ4 and INQ5 (Table 4). Item misfit was found for the items INQ2 and INQ5 (see S6 Table in the S1 Material). Contrary to the clinical and general population samples it was not possible to establish fit to a GLLRM–even after accounting for the sample DIF and the LD. While there was no more evidence of departures from the GLLRM in form of lack of homogeneity, further DIF or item misfit (see Table 2, and the S4 and S6 Tables in the S1 Material), evidence of additional LD could be identified. Presumably, additional LD existed for the item pairs INQ2 and INQ4 as well as INQ3 and INQ6 (see S2 Table in the S1 Material). However, due to convergence problems a concluding evaluation was not possible. Reliability and targeting are not reported because of inadequate fit to the models.

Although, no final GLLRM could be established, it would be instructive to evaluate the size of the DIF related to sample with the non-final GLLRM model, which accounted for the sample-DIF of four items and the LD between three pairs of items. We found this proceeding feasible, as there was no evidence of additional DIF. The impact of the sample-DIF was obvious. The difference found for the equated scores ranged from .44 to 2.60 across the score range and was > 2 for most scores (see S11 Table in the S1 Material). In doing so, the values for the general population were systematically lower. In case of a group study being interested in comparing the mean scores of the general population vs. the clinical sample it would make a difference whether one accounted for the found DIF. For the present samples the difference in means would be 3.55 score points without the necessary DIF-adjustment whereas it would be 5.03 with adjustment (see S14 Table in the S1 Material).

### Summary for PB-subscale

Taken together, the results of the analyses indicated that after rescoring and adjusting for LD and age-related DIF in case of the clinical sample, fit to a GLLRM could be shown for the clinical and general population samples. The PB-scale was well targeted for a sample of patients being treated for either an affective disorder or because of suicidal thoughts or behavior. However, as expected, targeting of the PB scale was poor for the general population. Regarding measurement invariance across samples, four out of six items displayed DIF related to sample. The size of DIF was such that splitting for DIF is strongly recommended. This implicates that the PB scale doesn’t work the same in the clinical as compared to the general population.

### Item analysis according to the Rasch model: TB-subscale

Items 7, 8, 10, 13, 14, and 15 had to be reverse coded in order to ensure that all items of the scale were scored in the same direction with higher scores indicating higher levels of TB. In the clinical sample, being the target group of the scale, a unidimensionality test was performed. This test indicated that the six rescored items and the three other items did not form a unidimensional scale (expected γ = .535, observed γ = .356, sd = .039, asymptotic p < .000, exact p < .000). Therefore, the three negatively formulated items were excluded from further analyses. These three items were item 9 (“These days, I rarely interact with people who care about me”), 11 (“These days, I feel disconnected from other people”) and 12 (“These days, I often feel like an outsider in social gatherings”).

Nonetheless, fit to the RM could not be found for any of the four different samples for the TB-subscale. In the subsequently performed analyses with the remaining six items, the items exhibited reversed thresholds. Due to convergence problems it was not possible to complete GLLRM analyses. As with the PB-subscale, the response categories of all items were recoded in the same way as the PB-subscale.

Despite recoding, neither fit to the RM nor to an alternative GLLRM could be achieved for the clinical sample, when item 10 (“These days, I am fortunate to have many caring and supportive friends”) was included. As the clinical sample is the target group for the INQ, this item was excluded from analyses for all samples to ensure better comparability. Therefore, all reported analyses were performed with the remaining five items and with the recoded response categories.

### Clinical population

**Fig 2:**
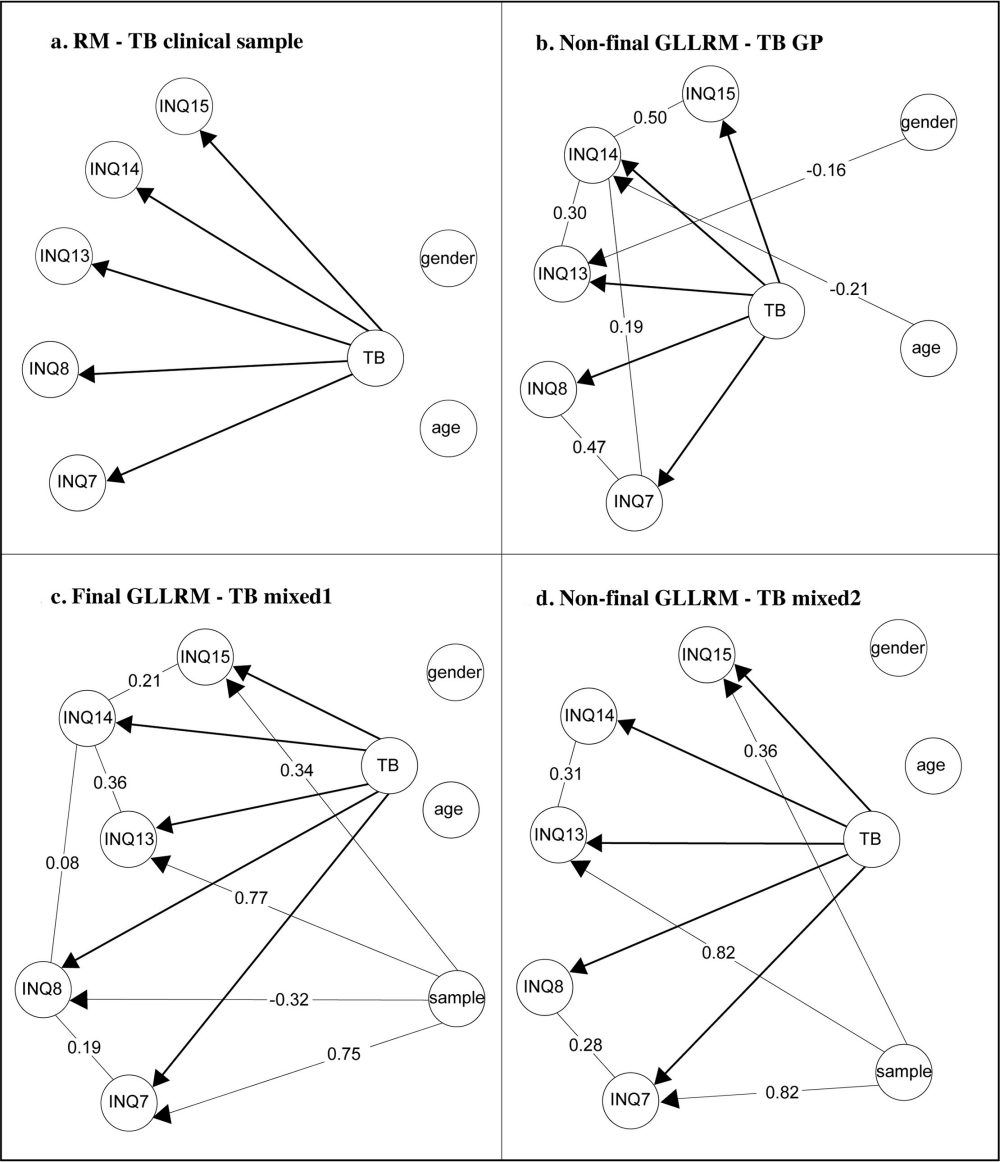
The Rasch model and graphical loglinear Rasch models for the TB-subscale. Lines between items show evidence of LD, arrows pointing from the exogeneous variables to the items evidence of DIF. Note. γ-correlations are partial Goodman and Kruskal’s rank correlations for ordinal data.

After recoding the response categories and eliminating the problematic item INQ10 from the TB-subscale, fit to the RM was found for the clinical sample (see Table 5, Fig 2), with no individual item misfit (see S7 Table in the S1 Material), and no evidence of DIF nor LD (see S3 and S5 Tables in the S1 Material).

**Table 5:** TB-subscale (4 response categories): Global test-of-fit and global tests-of-no-DIF relative to gender, age group and sample.

					DIF relative to									Comment
Sample	model	homogeneity			gender			age			sample			
		CLR	df	p	CLR	df	p	CLR	df	p	CLR	df	P	
*Clinical*	RM 6	38.9	17	**.002**^a^	20.3	17	.259	75.6	51	.014^a^				
	RM 5	11.3	14	.659	18.7	14	.178	63.1	42	.019^**b**^				
*GP*	RM	360.4	14	**< .001**	33.3	14	.003	70.3	42	**.004**^a^				No final model could be reached–indication of more LD and DIF relative to age
	GLLRM^§^	139.1	62	**< .001**	60.9	56	.304	196.0	150	**.007**^**a**^				
*Mixed1*	RM	31.0	14	**.006**^**a**^	9.5	14	.796	51.5	42	.150	118.3	14	**< .001**	
	GLLRM	47.1	40	.205	31.2	40	.840	159.5	120	**.009**^**c**^	36.3	22	.028^**c**^	
*Mixed2*	RM	46.5	14	**< .001**	15.5	14	.342	42.8	42	.438	127.3	14	**< .001**	No final model could be reached–indication of more LD
	GLLRM^§^	71.8	60	.140	26.9	26	.415	98.0	78	.062^a^	6.4	2	**.041**^a^	

**GP:** general population; **Clinical:** clinical sample. Benjamini-Hochberg adjustment for FDR

^a^ Reject if p-value is< .0500.

^b^ Reject if p-value is < .0167.

^c^ Reject if p-value is < .0125

^§^There was no final GLLRM.

The reliability and targeting were good, with 0.80 and with an average achievement of 81.5% of the maximum obtainable test information respectively (see S9 Table and S17a Fig in the S1 Material).

### General population

In the general population, the TB-subscale showed neither fit to the RM nor to a GLLRM (see Table 5). Departures from the RM were found in manifold form: There was evidence against global homogeneity and the overall test-of-no-DIF yielded strong evidence of DIF by age as well as gender (see Table 5). The age-related DIF was for item INQ14 (“These days, I am close to other people”), the gender-related DIF for item INQ13 (“These days, I feel that there are people I can turn to in times of need” (see Table 6)).

**Table 6:** Conditional likelihood ratio tests of DIF under the respective GLLRMs for the TB-subscale in the different samples.

Item and background variable	CLR	df	p
*GP*			
INQ13 & gender	18.19	3	**< .001**
INQ14 & age	25.41	9	**.002**
*Mixed1*			
INQ7 & sample	69.40	3	**< .001**
INQ13 & sample	58.11	3	**< .001**
INQ15 & sample	34.69	3	**< .001**
*Mixed2*			
INQ7 & sample	32.83	3	**< .001**
INQ8 & sample	14.51	3	**< .001**
INQ13 & sample	30.77	3	**< .001**
INQ15 & sample	31.96	3	**< .001**

**GP:** general population.

All items exhibited misfit, except item INQ7 (see S7 Table in the S1 Material). Moreover, several locally dependent pairs of items were found: INQ7 (“These days, other people care about me”) and INQ8 (“These days, I feel like I belong”), IN7 and INQ14 (“These days, I am close to other people”), INQ13 (“These days, I feel that there are people I can turn to in times of need”) and INQ14, and INQ14 and INQ15 (“These days, I have at least one satisfying interaction every day”) (see Table 7).

**Table 7:** Conditional likelihood ratio tests of local dependence under the respective GLLRMs for the TB-subscale in the different samples.

Items in subscales	CLR	Df	p
*GP*			
IN7&INQ8	534.73	9	**< .001**
IN7&INQ14	240.69	9	**< .001**
INQ13&INQ14	503.79	9	**< .001**
INQ14&INQ15	335.38	9	**< .001**
*Mixed1*			
IN7&INQ8	24.12	9	**.004**
INQ13&INQ14	39.03	9	**< .001**
*Mixed2*			
INQ7&INQ8	41.13	9	**< .001**
INQ8&INQ14	34.23	9	**< .001**
INQ13&INQ14	24.49	9	**.003**
INQ14&INQ15	26.59	9	**.001**

**GP:** general population.

However, even after accounting for the discovered DIF and LD, no fit to a GLLRM could be established (see Table 5). Although no evidence for age-related DIF for further items could be found (see S5 Table in the S1 Material), the global test-of-no-DIF still showed strong evidence for additional age-related DIF. The investigation of any further departures was not possible because of convergence problems (see S3 Table in the S1 Material). The targeting of the general population is not shown due to the inadequate fit to the models. However, a considerable floor effect was observed with 17.94% of the people having the lowest score in the TB-subscale

### Mixed1 sample of general population and clinical population

In the evaluation of the TB-subscale for the mixed1 sample, the RM was also rejected, with a significant global test-of-fit indicating no homogeneity of the item parameters as well as a significant global test-of-no-DIF, showing evidence of DIF related to sample (see Table 5). This DIF by sample was for items INQ7 (“These days, other people care about me”), INQ13 (“These days, I feel that there are people I can turn to in times of need”) and INQ15 (“These days, I have at least one satisfying interaction every day”) (see Table 6).

Furthermore, LD between two pairs of items was observed: items INQ7 and INQ8 (“These days, I feel like I belong”), and items INQ13 and INQ14 (“These days, I am close to other people”) (see Table 7). The item INQ14 was the only item exhibiting misfit (see S7 Table in the S1 Material). After accounting for these departures related to DIF and LD, fit to a GLLRM was obtained (see Table 5). Although DIF related to age was found in the global test-of-no-DIF, there was no evidence of additional DIF at the item level. Likewise, there was no indication of additional LD or item misfit (see Fig 2, and S3, S5 and S7 Tables in the S1 Material).

Finally, a reliability of .77 and .83 was observed for the clinical sample and the general population respectively, as were similar test information target indices for both subgroups of 0.82 indicating that 81.8% of the maximum obtainable test information was reached on average (see S17b Fig in the S1 Material).

The evaluation of the impact of the DIF related to sample showed that the score of the clinical sample was systematically higher than the score of the general population (see S12 Table and S15c Fig in the S1 Material). The difference found for the equated scores ranged from .32 to 2.63 across the score range. In case of a group study comparing the mean scores of both samples, the difference in means would be 1.12 score points without the necessary DIF-adjustment whereas it would be 3.04 with adjustment (see S14 Table in the S1 Material).

### Mixed2 sample of general population and clinical population

Fit to the RM could not be found in this second mixed sample. The global test-of-fit suggested no overall homogeneity and the global test-of-no-DIF suggested DIF related to sample (see Table 5). As in the mixed1 sample, items INQ7, INQ13 and INQ15 were found to have DIF relative to sample. Additionally, there was also evidence of sample-DIF for item INQ8 (see Table 6). Several pairs of items were locally dependent: INQ7 and INQ8, INQ8 and INQ14, INQ13 and INQ14, as well as INQ14 and INQ15 (see Table 7). And as in the mixed1 sample, misfit of INQ14 was observed (see S7 Table in the S1 Material). Despite accounting for these departures, it was not possible to establish fit to a GLLRM (see Table 5 and Fig 2). There was no more evidence for additional DIF (see S5 Table in the S1 Material). However, there was evidence for additional LD, which could, however, not be further investigated due to convergence problems. (see S3 Table in the S1 Material). Reliability and targeting are not reported because of inadequate fit to the models.

Even though, no final GLLRM could be established, the impact of DIF related to sample was estimated with the non-final model accounting for sample-DIF of four items and for LD of four items to get a rough impression of the magnitude. Noticeably, the mean of the general population sample of the mixed2 sample was lower than in the mixed1 sample. This indicated that the sense of belonging was more pronounced in the subgroup of the general population of the mixed2 sample (see S14 Table in the S1 Material). As with the mixed1 sample, the score of the clinical sample was systematically higher than the scores of the general population, when comparing the equated scores (see S14 Table and S15d Fig in the S1 Material). The difference found for the equated scores ranged from .32 to 2.40 across the score range.

### Summary for TB-subscale

After recoding the response categories, fit to the RM could be found for a 5-item version of the TB-subscale for the clinical sample. No fit to a RM nor to a GLLRM could be found for the general population and the mixed2 sample. The targeting of the TB-subscale was much better for the clinical sample than for the general population sample. In this sense, the TB-subscale like the PB-subscale is well targeted for a sample of patients being treated either for an affective disorder or because suicidal thoughts or behavior. In the mixed samples the uniform DIF related to sample was resolved. However, as with the PB scale the TB scale did not work the same way in the clinical sample as compared to the general population sample.

## Discussion

The present study is one of the few studies in applying the RM to evaluate the psychometric properties of the INQ and to refine its subscales (PB and TB) for use in clinical practice and research of predictors and correlates of suicidal ideation. Two aspects which have not been investigated in other studies before were of special interest: Applying the RM and the GLLRM made it possible to investigate the targeting of the INQ subscales, LD across items as well as the question whether the INQ subscales can be used the same way across different samples. The latter was investigated by analysis of DIF by sample in two mixed samples of patients and people from the general population.

Given the large sample size difference between the general population sample and the clinical sample, the different age structure in both samples and, given that little was known about whether PB and TB can be assessed in a uniform manner across the two different populations, several lines of analyses were carried through for the two INQ subscales. For both INQ subscales direct fit to the RM was not possible, and adjustments regarding the recoding of the response categories, the exclusion of problematic items and adjustments for LD and DIF, had to be applied. To ensure comparability across the different samples, the same adjustments related to the recoding of the response categories and the exclusion of items were made for each sample.

### PB-subscale

In summary, for the PB-subscale it was only possible to achieve fit to a GLLRM for the clinical sample and the general population sample with adjustment strategies such as the recoding of all items in four categories, and the inclusion of particular departures related to LD and DIF. In contrast, in the mixed1 sample fit to a GLLRM could not be established due to convergence problems. There was evidence of additional LD.

Overall, strong evidence of LD was found for all three samples, i.e. LD between items INQ1 and INQ2 was found for all samples. The further locally dependent item pairs observed in the clinical sample (INQ1 and INQ4) and in the general population sample (INQ4 and INQ5) were also found in the mixed1 sample. The LD could be related to item content, suggesting for example that the perceptions of burdensomeness represented with “better without me” and “happier without me” might have the same meaning for the people. The same applies for the item pair INQ4 “My death is a relief to others” and INQ5 “Wish they could be rid of me”, with both items involving the feeling of releasing family members and friends of the liability that the individual believes to represent.

The findings related to the LD of INQ1 and INQ2 are consistent with the results reported by Hallensleben et al. (2016), where the inclusion of residual correlations between these items in the CFA allowed to improve the fit of the obtained final model for this population. [11] Moreover, El-Behadli and colleagues suggested that items INQ1 and INQ2 as well as items INQ4 (in their study listed with the number 9) and INQ5 may capture very similar ideas, since they observed a strong correlation between these pairs of items in the evaluated clinical sample. [15]

DIF was investigated related to age and gender in all samples and additionally related to sample in the mixed1 sample. There was no evidence of DIF by gender in any of the samples. However, in the clinical sample age-DIF was found for of item INQ2 (“These days the people in my life would be happier without me”), although impact was rather low. The score difference at the same level of perceived burdensomeness was less than one score point. Nevertheless, the DIF by age should be further investigated in future patient studies. As the clinical sample of the present study was mainly made up of young and middle-aged adults (average age 37.87 years ± 13.07), it would be relevant for clinical purposes to investigate DIF by age in clinical samples that include a larger number of older adults, since in literature it has been suggested that these feelings of burden might be more present in older individuals. [43]

Regarding DIF related to sample, strong evidence of DIF was found for items INQ1, INQ2, INQ4 and INQ5. All four items were scored higher by the clinical sample given the same level of PB. Although the mixed1 sample data could not be fitted to a GLLRM, due to evidence of additional LD, which could not be modelled in this small sample, we found it relevant to evaluate the effect of the DIF. Particularly so, as there was no evidence of additional DIF. The DIF-equated scores showed the effect of DIF to be a difference of more than 2 score points for the majority of the scale. Thus, the INQ-items most probably were interpreted in different ways by the clinical and general population sample. Such a different interpretation of item content could be explained by cognitive distortions commonly experienced by patients suffering from suicidal ideation and/or affective disorders. [44] These cognitive distortions, defined as errors in cognitive processing as well in content of cognition, are assumed to play an integral role in the development and maintenance of suicidal ideation as well as of depression. [44] Among others, typical cognitive distortions found in patients with suicidal ideation and/or depression are cognitive rigidity, dichotomous thinking, overgeneralization, rumination and selective abstraction. Regarding the content of cognitions, it is often characterized by greater hopelessness and negative appraisal about the self and the future. Such cognitive vulnerabilities could lead to negative conclusions of oneself as “being a burden to others” and “others being happier without me”.

That the content of the items does not really reflect the way of thinking in the general population is also demonstrated by the extremely poor targeting of the PB-subscale. The target index was exceptionally low (.10) and the floor effect exceptionally high (86.6%). Contrary, the targeting for the clinical sample was appropriate.

### TB-subscale

Regarding the TB-subscale, four out of nine items had to be removed before fit to a RM or GLLRM could be established. This decision was based on the analysis of the clinical sample being the proper target group of the INQ. After the three negatively worded items were excluded (INQ9, INQ11 and INQ12), as they didn’t form a unidimensional scale with the other items, item INQ10 also had to be excluded. The response categories of the five remaining items (INQ7, INQ8, INQ13, INQ14 and INQ15) had to be recoded into four response categories. Applying these strategies, fit to the RM was established in the clinical sample. Thus, in the clinical sample the 5-item version of the TB-subscale is free of DIF related to gender and age and any LD. After adjusting for departures from the RM in form of LD and sample-DIF, fit to a GLLRM was achieved in the mixed1 sample. However, for the general population and the mixed2 sample neither fit to the RM nor to a GLLRM could not be established due to convergence problems.

The exclusion of the three negatively worded items INQ9, INQ11 and INQ12 had been done as the test of unidimensionality across the positively and negatively phrased items showed that these did not provide a unidimensional assessment of the TB construct. This is line with the results reported by Podlogar and colleagues. [45] Even though they did not use the RM approach, they also reported a better functioning of the TB-subscale when excluding the negatively worded items. Likewise, El-Behadli and colleagues had also excluded INQ9 in their study due to the item misfit. In contrast, INQ11 and INQ12 were retained in their final version because these items showed fit to the RM. However, considering the large percentage of unexplained variance in the first contrast of 12.3 reported by El-Behadli and colleagues for the TB-subscale, it is possible to suspect the existence of a second dimension in their refined version of this subscale. [46,47]

The exclusion of the INQ10 in our study coincides with the findings reported by El-Behadli et al. [15] They excluded INQ10 due to poor item discrimination, i.e. the item added little to the TB-subscale’s ability to measure the TB construct. [15]

Apart from the clinical sample for which fit to a RM could be found, the analyses of the general population and the mixed samples exhibited strong evidence of LD. In particular, the pairs of items INQ7 and INQ8 as well as of items INQ13 and INQ14, presented LD across these samples. A possible explanation for this response dependence, could be the similar information expressed in the items. Thus, item contents were related to the reciprocal care aspect (INQ7 “care about me” and INQ8 “I belong”) or the feeling of connection with others (INQ13 “people I can turn to” and INQ14 “close to other people”).

Just like for the PB-subscale strong evidence of DIF related to sample was found in the mixed samples. In both mixed samples DIF related to sample was found for items INQ7 (“These days, other people care about me”), INQ13 (“These days, I feel that there are people I can turn to in times of need”), and INQ15 (“These days, I have at least one satisfying interaction every day”) with the clinical sample presenting higher scores in these items compared to the people from the general population sample at the same level of thwarted belongingness. In doing so, it has to remembered that in case of the TB-subscale higher scores represent less of the feeling to belong. Interestingly, INQ8 (“These days, I feel like I belong”) displayed sample-DIF in the mixed1 sample with an opposite direction of the found DIF. Thus, for this item people from the general population sample scored higher. Overall, the extent of sample DIF was similar to the PB-subscale, which could again indicate a different perception of the content exposed by these items in the studied samples. [44] For both of the subscales it has to be remembered, that non-extreme people of the general population sample were included. So, the DIF effect between both samples indicating different interpretations of the item content might in total be even more pronounced.

The targeting of the TB-subscale was adequate for the clinical sample. The floor effect for the general population sample was not as pronounced as for the PB-subscale (20.5%). However, the targeting of the TB-subscale could only be investigated in the analysis of the mixed1 sample due to inadequate model fit found for the complete general population sample. For this subsample of the general population the target index was good (.82). However, this value probably doesn’t reflect the real quality of targeting as in the mixed1 sample only persons from the general population who belonged to the minority of people having non-extreme scores in the PB-subscale were included.

## Conclusion

Applying the Rasch model and the graphical loglinear Rasch model to the INQ-15, this study was supportive of some findings reported in previous studies using classical test theory-based methods. In general, the INQ subscales exhibited a good level of fit to the Rasch model requirements when used in clinical settings. Thus, for the clinical sample fit to a graphical loglinear Rasch model could be established for the PB-subscale. A 5-item version of the TB-subscale fitting a Rasch model was found. Both subscales were well targeted for use in clinical samples. The inclusion of items assessing the upper end of the constructs, i.e. a high level of perceived burdensomeness or thwarted belongingness, could contribute to assess these upper levels with higher measurement precision. However, our study did not support the findings from previous studies using classical test theory which reported an adequate performance of the questionnaire in non-clinical populations. [10,11] The results of our study strongly suggest, that the INQ subscales are not only poorly targeted for use in the general population, but that they might measure differently in the clinical setting as opposed to the general population.

Further research of the INQ under the Rasch approach in a larger and more diverse clinical sample is recommended to determine and optimize the item performance in these populations as well as to generalize the findings to other mental health conditions. It is also suggested that methods such as CFA should be applied in a larger clinical sample to assess the fit of the two factor structure of the INQ while taking into account the local dependence and differential item functioning evidenced in this study.

### Limitations

In the present study some limitations should be considered. First of all, the age structure was very different in both samples (general population and clinical sample). With the clinical sample being younger in average. Therefore, to exclude age-related effects when investigating sample-DIF age (and gender) matched mixed samples had to be used. Second, compared to the sample size of the general population, the size of the clinical sample was rather small. However, because of the shortness of the two subscales and because of the good targeting of the two subscales for patients diagnosed with either an affective disorder and/or with suicidal ideation, the size of the clinical sample was large enough to investigate the psychometric properties of the INQ. Nevertheless, future studies should include a larger and more diverse clinical sample. In doing so, it would also be relevant to investigate DIF related to the presence/absence of suicidal ideation. This would especially be informative for patients suffering from a depressive episode. As we argued, cognitive distortions are existent in both patient groups–patients with an affective disorder and/or suicidal ideation. Thus, it would be important to investigate whether patients with a depressive episode reporting suicidal ideation “just” differ in the extent of reporting PB or TB or whether they perceive these items differently (DIF) as compared to patients with a depressive episode not reporting suicidal ideation. Unfortunately, this information was missing for the sample with affective disorder in the present study.

## Supporting information

S1 Material (PDF)

S1 Data (XLSX)
