# Supplementary material for: Psychometric evaluation of the Interpersonal Needs Questionnaire (INQ) using item analysis according to the Rasch model
Source: PLoS One. 2020 Aug 3;15(8):e0232030. doi: 10.1371/journal.pone.0232030 (PMC7398530; doi:10.1371/journal.pone.0232030)
Supplement: S1 Material — (PDF) [file pone.0232030.s001.pdf]

Psychometric evaluation of the Interpersonal Needs Questionnaire (INQ) using item analysis according to the Rasch model

Supplementary Material

S1 Table. INQ-15 items 2

S2 Table. Conditional likelihood ratio tests of local independence under the GLLRM for all the items for the PB-subscale 4

S3 Table. Conditional likelihood ratio tests of local independence under the GLLRM for all the items for the TB-subscale 6

S4 Table. Conditional likelihood ratio tests of no DIF relative to gender, age and sample under the PB-subscale subscale GLLRMs 8

S5 Table. Conditional likelihood ratio tests of no DIF relative to gender, age and sample under the TB-subscale subscale GLLRMs 9

S6 Table. Item fit statistics comparing the observed and expected item-rest-score correlations under the models for the PB-subscale 11

S7 Table. Item fit statistics comparing the observed and expected item-rest-score correlations under the models for the TB-subscale 13

S8 Table. Targeting and Reliability for the PB-subscale 15

S9 Table. Targeting and Reliability for the TB-subscale 16

S10 Table. Equated scores showing the impact of DIF for the PB subscales in the clinical sample by age group 17

S11 Table. Equated scores showing the impact of DIF for the PB subscale in the mixed1 by sample 18

S12 Table. Equated scores showing the impact of DIF for the TB subscale in the mixed1 by sample 19

S13 Table. Equated scores showing the impact of DIF for the TB subscale in the mixed2 by sample 20

S14 Table. Comparison of observed and DIF-adjusted means of PB and TB scores in mixed samples subgroups affected by differential item functioning 21

S15 Fig. DIF-equated score graphics in samples subgroups affected by differential item functioning 22

S16 Fig. Item maps distribution for the PB-Subscale 23

S17 Fig. Item maps distribution for the TB-Subscale 24

S1 Table. INQ-15 items

Perceived Burdensomeness (PB-subscale)

Item code	Item description	Original Response Options						
		Not at all true for me			Some what true for me			Very true for me
		1	2	3	4	5	6	7
INQ1	These days the people in my life would be better off if I were gone							
INQ2	These days the people in my life would be happier without me							
INQ3	These days I think I am a burden on society							
INQ4	These days I think my death would be a relief to the people in my life							
INQ5	These days I think the people in my life wish they could be rid of me							
INQ6	These days I think I make things worse for the people in my life							

Thwarted Belongingness (TB-subscale)

Item code	Item description	Original Response Options						
		Not at all true for me			Some what true for me			Very true for me
		1	2	3	4	5	6	7
INQ7[R]	These days, other people care about me							
INQ8[R]	These days, I feel like I belong							
INQ9	These days, I rarely interact with people who care about me							
INQ10[R]	These days, I am fortunate to have many caring and supportive friends							
INQ11	These days, I feel disconnected from other people							

INQ12	These days, I often feel like an outsider in social gatherings							
INQ13[R]	These days, I feel that there are people I can turn to in times of need							
INQ14[R]	These days, I am close to other people							
INQ15[R]	These days, I have at least one satisfying interaction every day							

Note. [R] Reverse-coded items. The items marked in grey are the items not being included in the final version of the TB-subscale.

S2 Table. Conditional likelihood ratio tests of local independence under the GLLRM for all the items for the PB-subscale

PB-subscale				Comment
**Items**	**χ2**	**Df**	**p**	
Clinical				
INQ1&INQ3	18.34	9	.031	
INQ1&INQ5	14.80	9	.096	
INQ1&INQ6	9.91	9	.358	
INQ2&INQ3	12.00	9	.213	
INQ2&INQ4	15.15	9	.086	
INQ2&INQ5	13.70	9	.133	
INQ2&INQ6	16.72	9	.053	
INQ3&INQ4	20.85	9	.013	
INQ3&INQ5	22.64	9	.007	
INQ3&INQ6	18.33	9	.031	
INQ4&INQ5	16.32	9	.060	
INQ4&INQ6	10.28	9	.328	
INQ5&INQ6	18.80	9	.026	
GP				
INQ1&INQ3	11.63	9	.235	
INQ1&INQ4	15.94	9	.068	
INQ1&INQ5	34.65	9	**.000**	
INQ1&INQ6	15.71	9	.073	
INQ2&INQ3	13.76	9	.131	
INQ2&INQ4	13.99	9	.122	
INQ2&INQ5	7.09	9	.627	
INQ2&INQ6	11.00	9	.275	
INQ3&INQ4	18.80	9	.026	

INQ3&INQ5	25.82	9	**.002**	
INQ3&INQ6	19.61	9	.020	*** No convergence. Delta = 10.7201
INQ4&INQ6	19.13	9	.024	
INQ5&INQ6	12.47	9	.188	
Mixed1				
INQ1&INQ5	15.57	9	.076	*** No convergence. Delta = 2.2515
INQ1&INQ6	17.96	9	.035	
INQ2&INQ3	23.77	9	**.004**	
INQ2&INQ4	22476.76	9	.000	*** No convergence. Delta = 131.7279
INQ2&INQ5	9.84	9	.363	
INQ2&INQ6	20.45	9	**.015**	
INQ3&INQ4	11.42	9	.247	
INQ3&INQ5	42.85	9	.000	*** No convergence. Delta = 50.4802
INQ3&INQ6	24.42	9	**.003**	
INQ4&INQ6	5.34	9	.804	

Note. Benjamini-Hochberg correction for false discovery rate rejects (5% critical level) for PB-subscale: at 0.00208 for the clinical sample, at 0.00400 for the GP and at 0.02917 for the mixed sample. If γ is negative it is no evidence of local dependence.

S3 Table. Conditional likelihood ratio tests of local independence under the GLLRM for all the items for the TB-subscale

TB-subscale				Comment
**Items**	**χ2**	**Df**	**p**	
Clinical				
INQ7&INQ8	16.86	9	.050	
INQ7&INQ13	8.44	9	.490	
INQ7&INQ14	12.97	9	.163	
INQ7&INQ15	14.28	9	.112	
INQ8&INQ13	9.70	9	.375	
INQ8&INQ14	13.95	9	.124	
INQ8&INQ15	16.32	9	.060	
INQ13&INQ14	16.11	9	.064	
INQ13&INQ15	5.09	9	.826	
INQ14&INQ15	12.75	9	.174	
GP				
INQ7&INQ13	206649.26	9	.000	*** No convergence. Delta = 927.0000
INQ7&INQ15	50.31	9	.000	
INQ8&INQ13	126.00	9	.000	
INQ8&INQ14	33728.10	9	.000	*** No convergence. Delta = 942.3267
INQ8&INQ15	108.48	9	.000	
INQ13&INQ15	783.27	9	.000	*** No convergence. Delta = 250.4339
Mixed1				
INQ7&INQ13	11.07	9	.270	
INQ7&INQ14	15.74	9	.072	
INQ7&INQ15	16.98	9	.049	
INQ8&INQ13	9.97	9	.353	
INQ8&INQ14	14.23	9	.114	

INQ8&INQ15	16.45	9	.058	
INQ13&INQ15	5.86	9	.753	
INQ14&INQ15	22.56	9	.007	
Mixed2				
INQ7&INQ13	21.89	9	**.009**	
INQ7&INQ14	9.15	9	.423	
INQ7&INQ15	1.34	9	.998	*** No convergence. Delta = 6.4090
INQ8&INQ13	7.49	9	.585	*** No convergence. Delta = 2.0027
INQ8&INQ15	21.92	9	.009	*** No convergence. Delta = 20.8160
INQ13&INQ15	15.39	9	.080	*** No convergence. Delta = 15.0806

Benjamini-Hochberg correction for false discovery rate rejects (5% critical level) for TB-subscale: at 0.00250 for the clinical sample, at 0.00250 for the Mixed1, at 0.01667 for the Mixed2, and for the GP sample it was not reported due to the no convergence. If γ is negative it is no evidence of local dependence.

S4 Table. Conditional likelihood ratio tests of no DIF relative to gender, age and sample under the PB-subscale subscale GLLRMs

PB-subscale									
**Items**	**gender**			**age**			**sample**		
	**χ2**	**df**	**p**	**χ2**	**df**	**p**	**χ2**	**df**	**p**
Clinical									
INQ1	5.02	3	.170	20.55	9	.01			
INQ2	3.85	3	.278						
INQ3	2.54	3	.467	13.67	9	.134			
INQ4	1.65	3	.648	13.52	9	.140			
INQ5	1.72	3	.631	5.39	9	.799			
INQ6	4.07	3	.253	6.34	9	.705			
GP									
INQ1	2.56	3	.464	9.81	9	.366			
INQ2	1.57	3	.666	3.55	9	.938			
INQ3	4.88	3	.180	15.63	9	.075			
INQ4	2.73	3	.434	8.00	9	.533			
INQ5	2.14	3	.544	10.98	9	.277			
INQ6	0.86	3	.836	10.98	9	.277			
Mixed1									
INQ1	1.78	3	.620	9.51	9	.391			
INQ2	3.76	3	.288	17.28	9	.044			
INQ3	0.99	3	.804	19.06	9	.024	2.13	3	.548
INQ4	0.55	3	.908	13.03	9	.161			
INQ5	0.81	3	.846	13.03	9	.161			
INQ6	2.23	3	.525	1.87	9	.993	1.77	3	.629

Note. Benjamini-Hochberg correction for false discovery rate rejects (5% critical level) for PB-subscale: at 0.00455 for the clinical sample, at 0.00400 for the GP and at 0.00357 for the mixed sample.

S5 Table. Conditional likelihood ratio tests of no DIF relative to gender, age and sample under the TB-subscale subscale GLLRMs

TB Subscale									
**Items**	**gender**			**age**			**sample**		
	**$\chi 2$**	**df**	**p**	**$\chi 2$**	**df**	**p**	**$\chi 2$**	**df**	**p**
Clinical									
INQ7	6.23	3	.101	13.64	9	.135			
INQ8	4.57	3	.206	12.52	9	.185			
INQ13	2.76	3	.430	18.97	9	.025			
INQ14	4.53	3	.209	12.06	9	.209			
INQ15	2.54	3	.467	7.90	9	.544			
GP									
INQ7	3.52	3	.317	15.06	9	.089			
INQ8	2.54	3	.468	5.97	9	.743			
INQ13				16.08	9	.065			
INQ14	6.25	3	.099						
INQ15	2.60	3	.458	9.60	9	.384			
Mixed1									
INQ7	2.02	3	.568	11.30	9	.256			
INQ8	3.23	3	.357	13.10	9	.158	6.27	3	.099
INQ13	2.10	3	.551	23.31	9	.005			
INQ14	0.66	3	.882	8.72	9	.463	7.85	3	.049
INQ15	2.68	3	.443	3.23	9	.954			
Mixed2									
INQ7	5.46	3	.141	8.73	9	.462			
INQ8	2.71	3	.438	10.97	9	.277			
INQ13	3.12	3	.374	17.99	9	.035			
INQ14	3.64	3	.303	7.77	9	.557	6.39	3	.094

INQ15	1.21	3	.749	5.34	9	.804			

Benjamini-Hochberg correction for false discovery rate rejects (5% critical level) for TB-subscale: at 0.00250 for the clinical sample, at 0.02143 for the GP sample, 0.00250 at for the Mixed1 and 0.00714 at for the Mixed2.

S6 Table. Item fit statistics comparing the observed and expected item-rest-score correlations under the models for the PB-subscale

Items	Item-restscore correlations								
	Fit to RM			Fit to final GLLRM			FIT to non-final GLLRM		
	Observed γ	Expected γ	p	Observed γ	Expected γ	p	Observed γ	Expected γ	p
Clinical									
INQ1	.780	.736	.246	.780	.823	.189			
INQ2	.841	.735	.005	.841	.813	.379			
INQ3	.706	.735	.446	.706	.681	.565			
INQ4	.817	.745	.068	.817	.763	.183			
INQ5	.666	.743	.070	.666	.694	.562			
INQ6	.699	.738	.304	.699	.686	.763			
GP									
INQ1	.965	.964	.929	.965	.972	.086			
INQ2	.979	.965	.011	.979	.976	.409			
INQ3	.948	.964	.003	.948	.954	.429			
INQ4	.979	.965	.014	.979	.968	.034			
INQ5	.955	.965	.095	.955	.966	.056			
INQ6	.962	.965	.636	.962	.954	.226			

Mixed1							
INQ1	.747	.681	.038		.780	.823	.189
INQ2	.783	.680	.002		.841	.813	.379
INQ3	.626	.682	.066		.706	.681	.565
INQ4	.780	.715	.053		.817	.763	.183
INQ5	.557	.701	.000		.666	.694	.562
INQ6	.694	.680	.660		.699	.686	.763

Note. RM= Rasch model. GLLRM=graphical loglinear Rasch model. γ = Goodman & Kruskal's gamma coefficients. Benjamini-Hochberg adjusted critical levels at 5% were for the RM: 0.01389 for the clinical sample, 0.01944 for the GP, 0.01944 for the Mixed1 sample. Benjamini-Hochberg adjusted critical levels at 5% were for the final GLLRM: 0.00526 for the clinical sample and 0.00250 for the GP. Benjamini-Hochberg adjusted critical level at 5% was for the non-final GLLRM of the Mixed1 sample: 0.00526.

S7 Table. Item fit statistics comparing the observed and expected item-rest-score correlations under the models for the TB-subscale

Item-restscore correlations									
	Fit to RM			Fit to final GLLRM			FIT to non-final GLLRM		
**Items**	**Observed γ**	**Expected γ**	**p**	**Observed γ**	**Expected γ**	**p**	**Observed γ**	**Expected γ**	**p**
Clinical									
INQ7	.048	.573	.092						
INQ8	.584	.563	.712						
INQ13	.579	.574	.935						
INQ14	.705	.564	.012						
INQ15	.568	.576	.881						
GP									
INQ7	.783	.773	.342				.783	.808	.018
INQ8	.802	.776	.009				.802	.759	.000
INQ13	.750	.775	.016				.750	.748	.839
INQ14	.871	.778	.000				.871	.854	.060
INQ15	.750	.776	.010				.750	.759	.435
Mixed1									

INQ7	.557	.619	.088	.557	.599	.273			
INQ8	.680	.617	.081	.680	.637	.217			
INQ13	.603	.620	.641	.603	.628	.501			
INQ14	.734	.621	.002	.734	.667	.058			
INQ15	.585	.619	.331	.585	.581	.927			
Mixed2									
INQ7	.634	.666	.334				.634	.620	.709
INQ8	.673	.671	.957				.673	.690	.599
INQ13	.662	.665	.921				.662	.632	.395
INQ14	.783	.668	.000				.783	.771	.673
INQ15	.668	.675	.809				.668	.669	.989

Note. RM= Rasch model. GLLRM= graphical loglinear Rasch model. γ = Goodman & Kruskal's gamma coefficients. Benjamini-Hochberg adjusted critical levels at 5% were for the RM: 0.00278 for the clinical sample, 0.03333 for the GP, 0.01000 for the Mixed1 sample and 0.01000 for the Mixed2 sample. Benjamini-Hochberg adjusted critical level at 5% was for the final GLLRM of the Mixed1 sample: 0.00294. Benjamini-Hochberg adjusted critical levels at 5% were for the non-final GLLRM: 0.01333 for the GP sample and 0.00333 for the Mixed2 sample.

S8 Table. Targeting and Reliability for the PB-subscale

Subscale and Samples with DIF subgroups	Theta						Sum score				Extreme scores	
	TI mean	TI max	TI target index	RMSE mean	RMSE min	RMSE target index	target	mean	Mean SEM	r	Lowest	Highest
Clinical											13	8
Agegroup ≤26	2.656	3.508	.757	.625	.630	.847	10.13	9.02	1.60	.89	0	4
Agegroup 27 to 36	2.769	3.684	.752	.635	.521	.820	7.20	7.27	1.63	.87	3	1
Agegroup 37 to 48	2.748	4.336	.629	.648	.479	.738	10.53	6.44	1.61	.88	5	1
Agegroup ≥49	2.337	3.888	.601	.716	.507	.709	6.05	7.91	1.46	.93	5	2
GP	.278	2.728	.102	3.648	.605	.166	8.88	.44	.32	.86	2170	2

Note. **GP:** general population; **Clinical:** clinical sample. **TI:** test Information**.**

S9 Table. Targeting and Reliability for the TB-subscale

Subscale and Samples with DIF subgroups	Theta						Sum score				Extreme scores	
	**TI mean**	**TI max**	**TI target index**	**RMSE mean**	**RMSE min**	**RMSE target index**	**target**	**mean**	**Mean SEM**	**r**	**Lowest**	**Highest**
Clinical	2.506	3.073	.815	.645	.570	.884	7.45	8.33	1.57	.80	1	6
Mixed1											8	14
Clinical	2.836	3.465	.818	.610	.537	.881	8.81	8.33	1.67	.77	1	6
GP	2.338	2.860	.818	.657	.591	.900	8.31	7.21	1.51	.83	7	8

Note. **GP:** general population; **Clinical:** clinical sample. **TI:** test Information.

S10 Table. Equated scores showing the impact of DIF for the PB subscales in the clinical sample by age group

Score	Age group			
	≤26 years	27 to 36 years	37 to 48 years	≥49 years
1	1.00	1.13	1.12	1.12
2	2.00	2.50	2.44	2.39
3	3.00	3.74	3.65	3.50
4	4.00	4.73	4.66	4.39
5	5.00	5.56	5.54	5.15
6	6.00	6.34	6.39	5.87
7	7.00	7.14	7.26	6.60
8	8.00	8.00	8.13	7.40
9	9.00	8.91	8.98	8.31
10	10.00	9.89	9.80	9.31
11	11.00	10.93	10.60	10.41
12	12.00	12.02	11.42	11.56
13	13.00	13.15	12.28	12.70
14	14.00	14.29	13.23	13.82
15	15.00	15.40	14.31	14.91
16	16.00	16.42	15.52	15.97
17	17.00	17.29	16.79	17.00

Note. GP: general population. **Clin:** clinical sample.

S11 Table. Equated scores showing the impact of DIF for the PB subscale in the mixed1 by sample

Score	Subgroup	
	Clin	GP
1	1.00	.56
2	2.00	1.01
3	3.00	1.43
4	4.00	1.92
5	5.00	2.55
6	6.00	3.40
7	6.00	3.40
8	8.00	5.58
9	9.00	6.67
10	10.00	7.70
11	11.00	8.69
12	12.00	9.68
13	13.00	10.71
14	14.00	11.83
15	15.00	13.14
16	16.00	14.68
17	17.00	16.41

Note. GP: general population. **Clin:** clinical sample.

S12 Table. Equated scores showing the impact of DIF for the TB subscale in the mixed1 by sample

	Subgroup	
**Score**	**Clin**	**GP**
1	1.00	.46
2	2.00	.90
3	3.00	1.39
4	4.00	1.97
5	5.00	2.67
6	6.00	3.49
7	7.00	4.39
8	8.00	5.37
9	9.00	6.43
10	10.00	7.63
11	11.00	8.99
12	12.00	10.53
13	13.00	12.15
14	14.00	13.68

Note. GP: general population. **Clin:** clinical sample

S13 Table. Equated scores showing the impact of DIF for the TB subscale in the mixed2 by sample

Score	Subgroup	
	Clin	GP
1	1.00	.24
2	2.00	.51
3	3.00	.90
4	4.00	1.60
5	5.00	2.79
6	6.00	4.14
7	7.00	5.31
8	8.00	6.39
9	9.00	7.47
10	10.00	8.60
11	11.00	9.79
12	12.00	11.04
13	13.00	12.34
14	14.00	13.68

Note. GP: general population. **Clin:** clinical sample

S14 Table. Comparison of observed and DIF-adjusted means of PB and TB scores in mixed samples subgroups affected by differential item functioning

Subscales and DIF-groups	Observed scores		Adjusted scores		
	Mean	SE	Mean	SE	Bias
PB subscale					
Mixed1, Clin	7.72	.37	7.72	.27	.00
Mixed1, GP	4.17	.37	2.69	.24	1.48
TB subscale					
Mixed1, Clin	8.33	.27	8.33	.27	.00
Mixed1, GP	7.21	.28	5.29	.29	1.92
Mixed2, Clin	8.33	.27	8.33	.27	.00
Mixed2, GP	5.38	.27	3.77	.28	1.61

Note. SE: Standard error.

S15 Fig. DIF-equated score graphics in samples subgroups affected by differential item functioning

PB subscale

S15a Fig. Clinical, age group

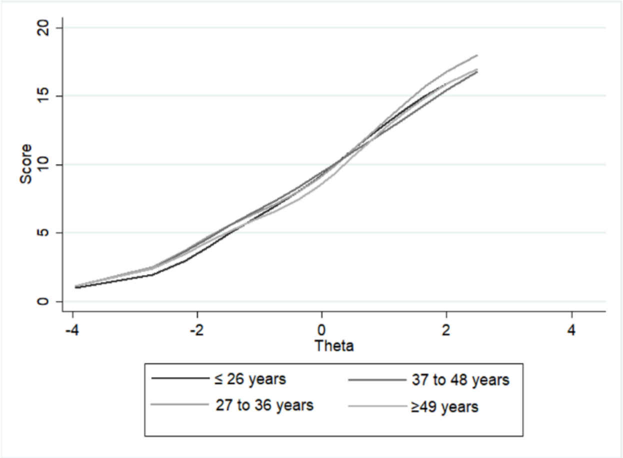

S15b Fig. Mixed1, sample

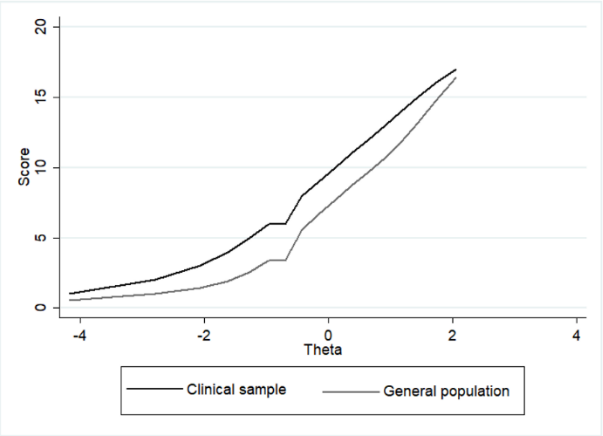

TB subscale

S15c Fig. Mixed1, sample

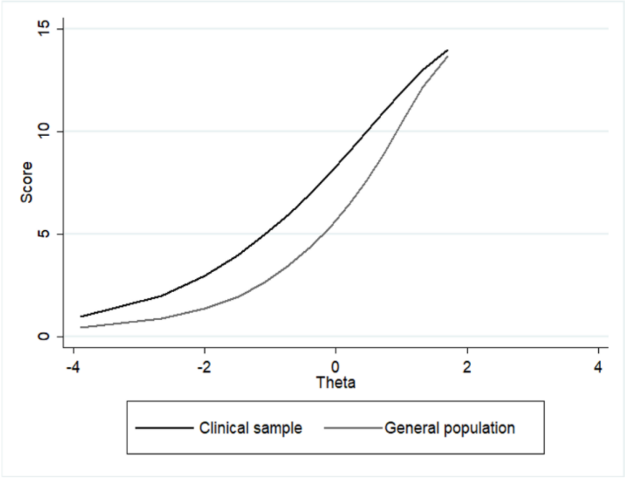

S15d Fig. Mixed2, sample

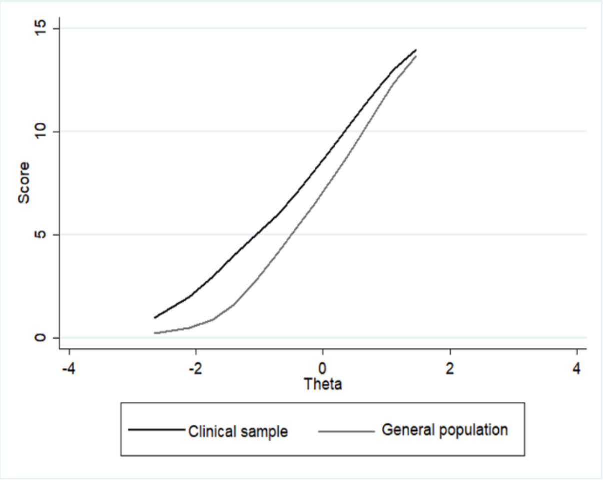

Note. This graphic shows the impact of DIF across the latent variable for the found DIF. The x-axis represents the latent construct with higher values indicating a higher perceived burdensomeness or thwarted belongingness respectively. For both subscales the clinical sample scored systematically higher given the same person parameter.

S16 Fig. Item maps distribution for the PB-Subscale

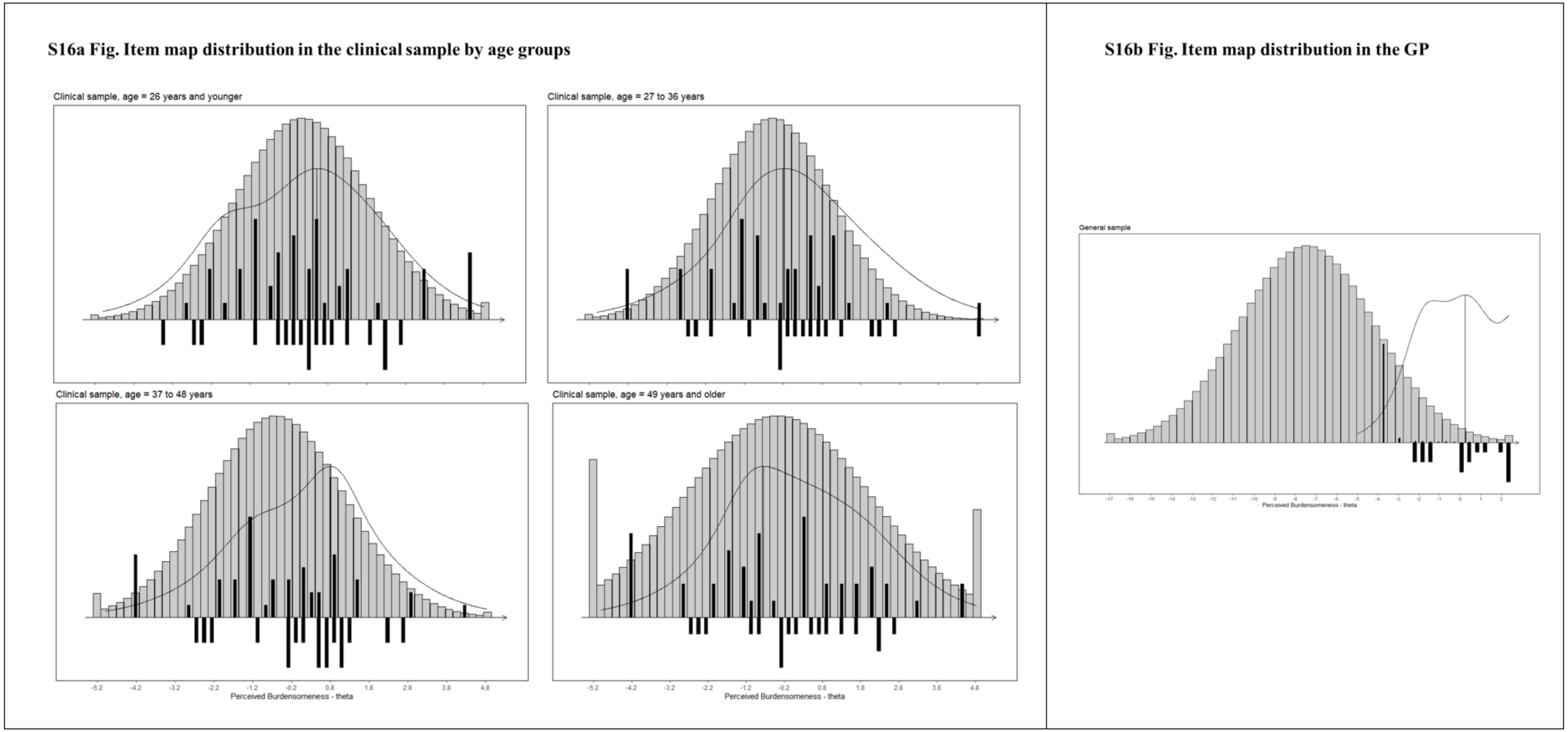

S16 Fig. Items maps with distributions of person parameter locations and information curve above item threshold locations. Note. The item maps illustrate how person parameters for the study sample (black bars above the line) and item threshold locations (black bars below the line) are distributed along the PB-dimension, with higher values indicating higher perceived burdensomeness (persons) and higher difficulty (item threshold) respectively. The person parameters are weighted maximum likelihood estimates. The grey bars above the line illustrate the distribution for the population under the assumption of normality. The black line represents the information curve, relative to the distribution of the item thresholds. For the clinical sample four item maps are shown for the subgroups for which evidence of age-DIF was found.

S17 Fig. Item maps distribution for the TB-Subscale

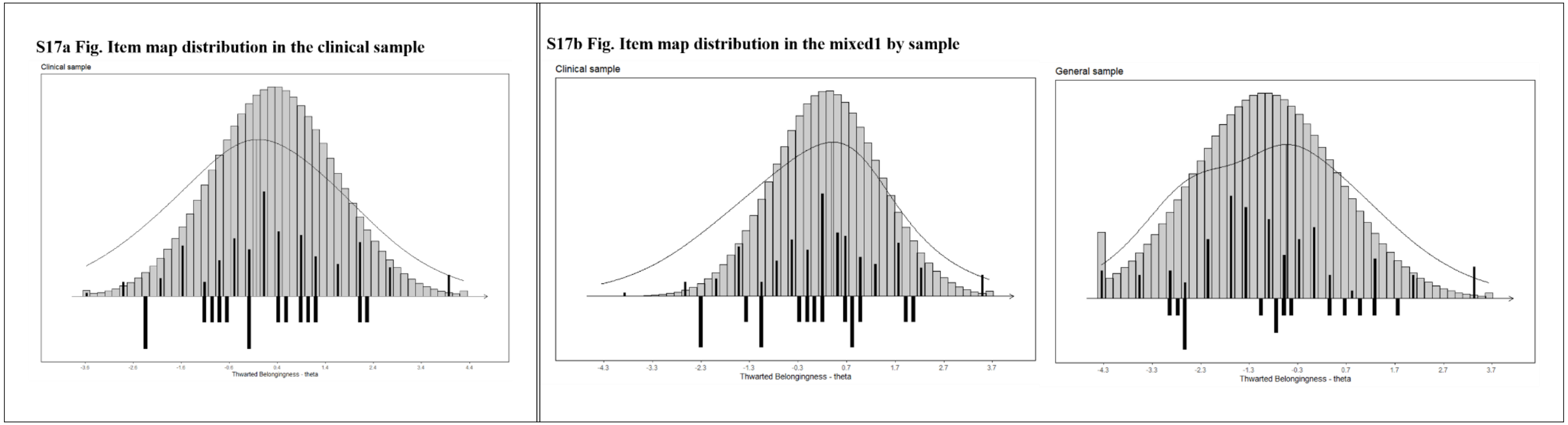

S17 Fig. Items maps with distributions of person parameter locations and information curve above item threshold locations. Note. The item maps illustrate how person parameters for the study sample (black bars above the line) and item threshold locations (black bars below the line) are distributed along the TB-dimension, with higher values indicating higher thwarted belongingness (persons) and higher difficulty (item threshold) respectively. The Person parameters are weighted maximum likelihood estimates. The grey bars above the line illustrate the distribution of these for the study sample (black bars above the line) and for the population under the assumption of normality. (grey bars above the line). The black line represents the as well as the information curve, relative to the distribution of the item thresholds. (black bars below the line). The vertical line from the information curve denote the point of maximum information. For the mixed1 sample, both subscales two item maps are shown in for the subgroups for which evidence of sample-DIF was found.
